# Supplementary material for: Are carbon emissions trading and green financial instruments synergistic? -Comprehensive quantitative research based on content analysis
Source: PLoS One. 2024 Mar 7;19(3):e0298601. doi: 10.1371/journal.pone.0298601 (PMC10919601; doi:10.1371/journal.pone.0298601)
Supplement: S1 Data — (ZIP) [file pone.0298601.s001.zip › Data/地级市Z_F工作报告.zip!/─┌├╔╣┼/░═╤σ─╫╢√╩╨/└┤╫╘╣1⁄2╓┌║┼í╛┬φ┐╦╩2╛▌═°í┐_2016.docx]

各位代表：

现在，我代表市人民政府向大会报告工作，请连同《巴彦淖尔市国民经济和社会发展第十三个五年规划纲要(草案)》一并审议，并请市政协委员和列席会议的同志们提出意见。

一、“十二五”时期及2015年工作回顾

“十二五”时期，是我市发展进程中具有里程碑意义的五年。在市委的坚强领导下，我们大力弘扬总干精神，以“迎庆创城”为总抓手，奋力开创改革发展新局面。特别是党的十八大以来，全市上下深入学习贯彻习近平总书记系列重要讲话和考察内蒙古重要讲话精神，全面落实自治区“8337”发展思路，守望相助，团结奋斗，全面建成小康社会迈出坚实步伐，全面深化改革实现良好开局，全面依法治市开启新征程，全面从严治党取得新进展，巴彦淖尔站在了新的历史起点上。

“十二五”时期，我们主动顺应形势任务新要求，调整优化发展思路，在赶超崛起的道路上实现了精彩跨越。面对发展环境的深刻变化，不断深化市情认识，创新发展思路，提出了建设富裕、美丽、幸福、文明、活力巴彦淖尔的发展愿景，全市上下凝心聚力，砥砺前行，基本完成了“十二五”确定的各项任务。预计 2015年实现地区生产总值890亿元、公共财政预算收入65.76亿元，分别是“十一五”末的1.5倍和1.7倍，社会消费品零售总额完成234亿元，比“十一五”末翻了近一番，固定资产投资累计完成3743亿元，是“十一五”时期的1.9倍，城乡居民收入分别达到24654元、13729元，均是“十一五”末的1.7倍，各项事业都上了一个大台阶。连续两年高水平接受了自治区巡回观摩检查，多次承办国家和自治区级赛事活动，巴彦淖尔对外形象和影响力大幅提升360小说。

“十二五”时期，我们主动适应经济发展新常态，坚持走绿色发展之路，可持续发展的基础更加坚实。准确把握新常态下速度变化、结构优化、动力转换等特点，注重产业转型升级，注重做好水、绿、文化三篇文章，注重为长远发展打基础、增后劲。绿色特色农牧业金字招牌越来越亮，肉羊出栏量、有机原奶产量、农畜产品出口额均居自治区首位，巴美肉羊成为国内第一个具有自主知识产权的肉羊品种。化工、冶金和生物医药等产业加快发展，清洁能源基地初步成型，风光电装机累计达到364万千瓦，占电力总装机的63%。黄河旅游精彩亮相，乘船游二黄河由梦想变为现实，第三产业增加值提高6.5个百分点。套区森林覆盖率达到 20%，新增湿地10万亩。严守生态环保红线，将污染项目拒之门外，保护了这方净土，我市成为全国首批生态文明先行示范区。城乡面貌焕然一新，累计完成城建投资966亿元，是“十一五”时期的2倍。累计争取到棚户区改造贷款84亿元，实施棚户区改造5.7万户;投入农村牧区危房改造资金42.3亿元，完成危房改造11.2万户，新农村新牧区建设走在全区前列。基础设施建设水平全面跃升。巴彦淖尔机场通航运营，乌中旗通用机场试飞成功。投资15.5亿元建成了279公里黄河堤防公路，投资7.5亿元的金川大桥和投资5.6亿元的临河黄河大桥建成，新改建公路8530 公里，新增铁路346公里，交通完成投资185亿元，乌前旗获评全国农村公路建设先进集体。电力完成投资近60亿元，首座百万千瓦风电汇集站建成投运。投资68.3亿元实施了680万亩农田整治，投资17亿元实施了大型灌区续建配套与节水改造，投资15.6亿元完成了河套灌区三年排水改造工程。政府机构、行政审批、城管体制、农垦体制等改革稳步实施。向北开放取得历史性突破，甘其毛都口岸五年过货量达5411万吨，是“十一五”时期的3.3倍。

“十二五”时期，我们主动回应人民群众新期待，实施了一批民生实事，人民群众幸福感和获得感不断增强。各级财政用于民生的投入累计达到701.3亿元，是“十一五”时期的2.6倍。新增城镇就业6.8万人，农牧民转移就业82.8万人次。养老、医疗保险实现制度全覆盖，企业退休人员养老保障水平不断提升，城乡低保应保尽保，16.7万贫困人口稳定脱贫。城乡居民医疗保险报销比例分别提高到75%、76.5%。投资12亿元、20万平方米的新区市医院加紧建设，市传染病院恢复启用。全面实施了十二年免费教育，投资21亿元新改扩建和加固校舍105万平方米，河套学院升本成功，占地1080亩的新校区即将完工投用。大力推进百姓安居工程，建成保障性住房4万套。全面实施安全饮水工程，解决了17万人安全饮水问题。新建自来水厂2座，形成黄河水、地下水两套供水系统，临河城区供水保障能力大幅提升。建成文博中心、黄河水利文化博物馆等公共文化设施，创作推出电视剧《我叫王土地》等优秀文艺作品，阴山岩刻进入世界文化遗产预备名单，乌后旗蒙古族驼球列入国家级非物质文化遗产名录。平安建设不断深化，社会治理扎实推进，人民群众安全感、满意度持续提升。中国梦和社会主义核心价值观深入人心，政风行风社风明显转变，干部群众精神面貌发生深刻变化，勤劳智慧的巴彦淖尔人正满怀信心创造美好生活。

刚刚过去的2015年，是“十二五”的收官之年，也是辛劳的一年、奋斗的一年、难忘的一年。我们积极应对经济下行压力，统筹推进各项工作，经济社会发展呈现稳中有进的态势。预计地区生产总值增长7.8%，固定资产投资增长14.4%，公共财政预算收入增长6%，社会消费品零售总额增长8.5%，城乡居民收入分别增长9%和10%。节能减排完成年度目标任务。

(一)“十个全覆盖”工程深得民心。把“十个全覆盖”工程作为最大的民生工程，举全市之力扎实推进。各地各部门多方整合资源，扩面提标，突出特色，探索出了切合实际的模式和做法。自治区督导组真情投入，帮助解决了大量困难和问题。2.3万名干部带着感情和责任进村入户示范引领，广大农牧民真诚拥护、主动参与、共建家园。各级干部群众顽强奋战、合力攻坚，冬闲时节也不停顿，继续开展清“五堆”、搞规划、备物料等工作。4271个村庄已有1764个实现全覆盖，1268个正在建设，“十个全覆盖”攻坚之战取得阶段性胜利，巴彦淖尔再次以独特的魅力展现在全区面前，自治区巡回观摩检查组给予了极高的评价。 “十个全覆盖”工程的实施，让农牧区更绿了、更美了、更水灵了，农牧民更幸福、更文明、更有奔头了，各级干部受到了教育、经历了锤炼、增强了本领，全市上下的自信心、自豪感再次被激发，干事创业的激情再次被点燃。

(二)产业发展态势稳中向好。粮食总产量达到69亿斤，实现“十二连增”。设施农业累计达到24万亩，肉羊饲养量2139万只，出栏1143万只。新增“三品”认证59个，河套面粉、圣牧高科有机奶获国际农交会金奖。预计规模以上工业增加值增长9.2%。华拓1500万吨铁矿石干选、东矿100万吨铅锌硫采选、蒙羊牧业100万只肉羊加工等项目建成投产。新增风电装机60万千瓦、光电装机14万千瓦。黄河河套文化旅游区“三带五区”初具规模，富强村、匈奴城、兵团博物馆、河套农耕文化博览苑、乌拉特部落水世界等旅游项目建成运营，公田村、民建村被评为中国最美休闲乡村，春光村、联丰村、海子沿村等7个村荣获中国乡村旅游模范村称号，乡村游、沙漠游、水上游、冰雪游正在兴起。全年接待游客352.4万人次，实现旅游收入35.2亿元。巴彦淖尔陆港通关运营，口岸物流园区被列为自治区服务业聚集区，建成2个国家级外贸转型示范基地。甘其毛都口岸进口煤炭609万吨、铜精粉87.6万吨，出口电力10.9亿度。进出口总额达到29亿美元，其中农畜产品出口4亿美元，增长25%。电子商务蓬勃兴起，葵花籽电子交易中心交易额累计达508亿元，羊畜产品电子交易平台交易额达1.5亿元，五原县成为“全国电子商务示范县”。市中小微企业融资担保公司成立，20家企业进入资本市场，直接融资9.7亿元。

(三)重要领域改革扎实开展。总书记提出的三项改革有序推进，编制完成了森林、草原、湿地资源实物量表，实施了领导干部自然资源资产责任审计试点;参加首届中蒙博览会并取得积极成果，双方口岸试行联合监管，关检合作实行“三个一”通关模式;92 家龙头企业与农牧民建立了紧密型利益联结机制，农牧民合作社达到4266家，带动农牧户13万户。政府权力清单编制完成并向社会公布，公共资源交易改革走在全区前列。“三证合一”、“一照一码”全面推开，新增市场主体2.6万户、注册资本215亿元，分别增长61%、4.9%。财税、金融、户籍制度等改革稳步推进。土地确权登记颁证、草原确权承包试点基本完成，公立医院、扩权强县、农村综合改革等试点进展顺利，乌梁素海管理体制等自主改革有效实施。政治、文化、社会等各领域改革均取得新进展。

(四)基础设施条件持续改善。城建投资完成152.2亿元。金川南路配套工程完工，先锋新桥加快建设，改造城镇出入口9个、老旧小区53万平方米，整治背街小巷1000多条，公厕旱改水140座，新增集中供热面积194万平方米、各类管网130公里、天然气入户2万户，实施生活垃圾和污水处理试点村 40个。编制了25个专项规划，开展了7300公里地下管线探测和建档工作。完成林业生态建设90万亩，新增园林绿化8639亩。实施高标准农田建设 162.5万亩，积极创建全国国土资源节约集约模范市。临哈高速、临甘一级公路建设进展顺利，口岸重载公路、青乌公路建成通车，新增通村公路1200公里，实施农村牧区街巷硬化2500公里。包兰铁路沿线环境综合整治初战告捷。

(五)民生保障工作力度加大。在财力十分紧张的情况下，想方设法保障民生，各级财政用于民生的支出达176.1亿元，占公共财政预算支出的 74.5%。投入15.2亿元推进扶贫开发，3.1万贫困人口实现脱贫，乌梁素海周边等重点区域扶贫效果初显。开展城乡劳动者创业培训5024人，创业带动就业1.4万人。“三个一”民生实事惠及27万农牧户、1211名大学新生，零就业家庭实现动态清零。开工保障房2.5万套，解决了7560户回迁房延期交付和7000户房产证办理遗留问题。18所幼儿园完成新建改建，50所义务教育学校完成标准化建设，2个旗县通过国家义务教育均衡发展验收，磴口县成为全国“改薄”工作典型县。启动了居民健康卡“一卡通”，“健康保障小药箱”进牧户基本实现全覆盖。全国龙舟公开赛、沿黄公路自行车赛、中俄蒙阴山岩刻 “双年展”等活动影响力不断扩大，全国中学生田径锦标赛在杭锦后旗举办，27所学校入选国家级足球特色学校。“五城同创”扎实推进，成为全国文明城市提名城市。安全生产形势持续稳定好转，食品药品和质量监管不断强化，信访总量大幅下降。应急救援和立体化打防控体系进一步完善，刑事和治安案件分别下降 16.1%和26.3%。双拥共建成效明显，国防和军队建设受到中央军委领导高度评价。

(六)政府自身建设不断加强。依法行政取得新进展，我市首获地方立法权。严格执行重大行政决策法定程序，政府法律顾问制度实现全覆盖，“六五”普法全面完成，公职人员尊法学法守法用法观念不断增强。自觉接受人大依法监督和政协民主监督，政府班子主动上门征求人大、政协班子的意见和建议。办理人大代表建议113件、政协委员提案282件，办结率均为100%，满意率分别达到95%、94%。支持法院、检察院依法独立行使职权，重视做好统战、民族、宗教、群团工作，凝聚了强大正能量。行政效能显著提升，市政务中心被列为国家级社会管理和公共服务标准化建设试点。第九届嘎查村两委换届圆满完成。认真落实全面从严治党要求，政府党组建设和政府系统党的建设制度化水平得到提升。加大对为官不为、懒政怠政、“吃空饷”等问题的整治力度，群众路线教育实践活动成果进一步巩固，“三严三实”专题教育成效明显。审计、监察和政务公开扎实开展，反腐倡廉建设深入推进。

各位代表，经过全市上下的共同努力，完成了全年主要目标任务，为“十二五”画上了圆满的句号。回首过去五年这段不平凡的历程，我们最振奋的是总干精神凝魂聚气、不断升华，最欣慰的是老百姓的日子红红火火、蒸蒸日上，最自豪的是广大干部群众不甘落后、奋勇争先，最难忘的是面对重重困难，我们众志成城，用心血和汗水书写了巴彦淖尔的精彩故事。这些成绩是市委总揽全局、科学决策的结果，是人大、政协加强监督、鼎力支持的结果，更是全市各族干部群众齐心协力、攻坚克难的结果。在此，我代表市人民政府，向所有关心支持巴彦淖尔建设与发展的同志们、朋友们，表示崇高的敬意和衷心的感谢!

成绩属于过去。面向未来，我们清醒地看到，我市发展仍面临诸多困难和挑战。主要是：经济下行压力依然存在，经济总量小，结构不合理，产业层次低、链条短、竞争力不强;大企业大项目少，企业创新能力不足，部分产品效益下滑;小微企业资本少、底子薄，发展艰难;财政增收十分困难，收支矛盾突出。生态环境还比较脆弱，基础设施建设历史欠账较多。公共服务水平不高，教育、医疗、养老、就业、金融服务和物业管理等方面还有许多群众不满意的地方。社会治理能力与现代社会发展的要求还有较大差距，重管理轻服务还没有根本转变，少数公职人员思想保守，工作应付，不担当、不作为、不勤不廉，社会上不诚信、不文明、不守纪等不良风气依然存在。我们必须增强忧患意识、责任意识，更加扎实有效地做好各项工作。二、“十三五”时期的目标任务及2016年工作

“十三五”时期是全面建成小康社会、实现第一个百年目标的决胜阶段。当前，我国经济发展进入新常态，仍处于大有可为的重要战略机遇期。党的十八届五中全会系统阐述了“五大发展理念”，开启了关系全局的一场深刻变革。国家和自治区深入实施新一轮西部大开发、“一带一路”、“中国制造2025”、经济结构性改革、“互联网+”、脱贫攻坚、法治政府建设等重大战略举措，为我们带来了难得的历史发展机遇。我市丰富的水土光热资源、矿产资源，以及交通区位、向北开放、人文环境等优势凸显，干部群众崇尚实干、争先进位的热情高涨。我们一定要顺应大势，抢抓机遇，主动作为，努力破解发展难题，厚植发展优势，不断开拓发展新境界。

“十三五”时期我市发展的指导思想是：高举中国特色社会主义伟大旗帜，全面贯彻党的十八大和十八届三中、四中、五中全会精神，以马克思列宁主义、毛泽东思想、邓小平理论、“三个代表”重要思想、科学发展观为指导，深入贯彻习近平总书记系列重要讲话和考察内蒙古重要讲话精神，坚持“四个全面”战略布局，坚持创新、协调、绿色、开放、共享发展理念，加快形成引领经济发展新常态的体制机制和发展方式，大力弘扬总干精神，做好“水、绿、文化”三篇文章，建设绿色农畜产品生产加工输出、清洁能源输出、有色黑色金属和进口资源深加工、现代化工及新材料生产、河套文化特色旅游度假基地，统筹推进经济、政治、文化、社会、生态文明建设，确保如期全面建成小康社会，着力构建富裕、文明、绿色、幸福巴彦淖尔。

根据市委“十三五”规划《建议》，市政府编制了“十三五”规划《纲要(草案)》，提出了我市未来五年发展的任务书和路线图，概括起来就是：奔向一个目标，激活两大动力，实现十个更加。

奔向一个目标：就是实现全面建成小康社会的总目标。经济增速不低于全区平均水平，能快就不要慢，努力实现有质量、有效益、没水分、可持续的发展，到 2020年地区生产总值和城乡居民收入比2010年翻一番。转变发展方式取得重大进展，民生福祉大幅增进，社会文明程度显著提升，生态环境质量持续改善，各方面体制机制进一步健全。

激活两大动力：一是深化改革。把改革作为推动发展的制胜法宝，努力在重点领域和关键环节取得实质性突破，释放新需求，创造新供给，增强新动能。二是创新创业。把创新作为引领发展的第一动力，不断推进各方面创新，促进新技术、新产业、新业态蓬勃发展，让大众创业、万众创新蔚然成风。

实现十个更加：一是产业更加壮大。“五个基地”初具规模，产业向中高端迈进，多元发展多极支撑的现代产业体系基本建立。二是城乡更加协调。打造自治区西部区域性中心城市，持续推进农村牧区“十个全覆盖”工程，城乡发展一体化新格局加快形成。三是基础更加完善。构建立体快捷的高速、高铁、航空交通体系和覆盖城乡的水、电、气、讯、路等现代基础设施网络。四是生态更加秀美。主要生态系统步入良性循环，初步建成全国生态文明示范区，为筑牢祖国北疆生态安全屏障做出更大贡献。五是开放更加活跃。建设向北开放桥头堡和融入“一带一路”重要枢纽，深化对内开放和区域合作，形成全面开放新格局。六是文化更加繁荣。建设民族文化大市，文化事业和文化产业加快发展，河套文化影响力持续扩大。七是收入更加殷实。居民收入增长高于经济增长，达到全区平均水平，现行标准下农村牧区贫困人口全部脱贫，自治区级贫困旗县全部摘帽。八是保障更加健全。公共服务体系进一步完善，就业、教育、社保、医疗、住房等基本公共服务均等化水平稳步提高。九是社会更加安定。全民共建共享的社会治理格局加快构建，祖国北疆安全稳定屏障不断巩固。十是作风更加务实。基本建成法治政府，全面提升政府执行力，以优良作风确保“十三五”规划落到实处。

各位代表，再奋力拼搏五年，到本世纪第二个十年结束的时候，我们将共同完成全面建成小康社会这项前无古人的伟大事业，巴彦淖尔这颗祖国北疆亮丽风景线上的耀眼明珠将绽放出更加灿烂的光彩。

2016年是全面建成小康社会决胜阶段的开局之年，也是推进结构性改革的攻坚之年。我们将按照“四个全面”战略布局和“五大发展理念”，积极适应经济发展新常态，以提高经济发展质量和效益为中心，以改革开放创新为动力，以加快产业转型升级为重点，着力加强供给侧结构性改革，坚持稳增长、调结构、惠民生、防风险，落实好“五大政策”和去产能、去库存、去杠杆、降成本、补短板“五大任务”，工作重点向“十个更加注重”转变，努力实现“十三五”良好开局。主要预期目标是：地区生产总值增长8%左右，固定资产投资增长10%左右，社会消费品零售总额增长10%，公共财政预算收入增长6%以上，城乡居民人均可支配收入分别增长9%和10%，完成节能减排和环境质量改善目标，推动结构性改革取得实质性进展。重点在十个方面下功夫：

(一)在抓好改革落地见效上下功夫。突出问题导向，完善落实机制，加强统筹协调，使改革不断见到实效，让群众有更多获得感。抓好三项重点改革。科学编制自然资源资产负债表，开展领导干部自然资源资产离任审计，实现排污权有偿使用和交易。深化与蒙方在矿能、电力、文化、旅游等领域合作，推进基础设施互联互通和贸易便利化。强化龙头企业联农带农与扶持政策挂钩激励机制，推广紧密型农企利益联结模式，让农牧民分享加工销售环节收益。落实国家供给侧结构性改革政策。引导企业开展兼并重组、技术改造、精深加工，扩大有效供给，稳妥化解产能过剩。通过正税清费、降低物流成本等多措并举，帮助企业减轻负担。推进户籍制度和住房制度改革，加快转移农牧民市民化进程，扩大公租房覆盖面，释放住房有效需求，促进房地产市场平稳健康发展。规范各类融资行为，防范化解金融风险。加大重要领域和关键环节改革力度。以简政放权、放管结合、优化服务为重点，持续推进政府职能转变，6月底前实现市级行政审批中介服务清单管理。7月底前编制完成市本级责任清单。完善公共资源交易平台，逐步实现异地远程评标和网上全程电子化招投标。深化财税体制改革，完善政府预算体系，加强税收征管，推进综合治税。深化投融资体制改革，推广政府与社会资本合作模式。加快金融体制改革，积极发展直接融资。深化农村土地制度改革，全面推开农村宅基地、土地确权登记颁证，做好临河区全国农村土地经营权抵押贷款试点工作。抓好供销社、国有林场、农业水价综合改革。扎实开展“扩权强县”、“多规合一”等改革试点。统筹推进其他改革。深化养老保险、教育、文化、信访、公安、司法、行政执法体制改革。创新社会治理体制，稳步实施事业单位、公交体制、社会征信制度改革。逐步建立统一的城乡居民基本医疗保险制度。深入推进医药卫生体制改革，推动县级公立医院改革政策落实到位。

(二)在激发全社会创新创业活力上下功夫。深入实施创新驱动发展战略，围绕重点领域和优势特色产业开展关键技术攻关，深化产学研合作，组织实施一批重点科技项目，加大科技成果转化力度。加强科技创新平台载体建设，创建国家农业科技园区、1-2家高新技术企业，新增2-3家自治区企业技术研发中心，专利授权150件以上。充分发挥河套学院、市农牧业科学院、河套农牧业技术研究院等科研机构作用，加快形成具有自主知识产权的科技创新成果。深入实施“河套英才”工程，加大人才培养使用引进力度。评选50名河套英才、10个创新创业团队，引进研究生学历或副高以上急需优秀人才150人。完善人才评价激励机制和服务保障体系，让人才来得了、留得住、用得好。实施“互联网+”行动计划，加快发展网络经济，推广新型孵化模式，鼓励发展众创、众包、众扶、众筹空间。新增城镇就业1.25万人，农村牧区劳动力转移就业14.5万人。全面落实支持创新创业的各项政策，发放创业担保贷款1.5亿元。实施高校毕业生就业促进和创业引领计划，开展农村牧区青年创业富民行动，创建自治区级、市级示范性创业园各1个。加大投资于人的力度，统筹用好1000万元创业就业发展基金，完成城乡职业技能培训2万人，使劳动者更好适应变化了的市场环境。大力支持河套学院、市职业技术学校培养复合型技能人才，建设市职业技能培训基地和示范性综合实践基地。深化商事制度改革。以敬民之心行简政之道，简化优化办事流程。完善政务服务网络，推广并联审批、网上审批，让信息多跑路、群众少跑腿。营造创业创新文化，让人们在创造财富的过程中，更好地实现精神追求和自身价值。

(三)在做精绿色有机农牧业上下功夫。围绕当好全区绿色农畜产品生产加工输出基地的排头兵，着力加强农牧业供给侧结构性改革，提高农牧业供给体系质量和效率，形成结构合理、保障有力的农畜产品有效供给。落实藏粮于地、藏粮于技战略，实施170万亩高标准农田建设、28万亩节水增效工程，粮食总产稳定在 60亿斤以上。争取480万亩改盐增粮项目纳入国家和自治区“十三五”规划，尽快启动实施。引导农民面向市场需求调整种植结构，实施控肥控药计划，划定绿色种植区域。制定有机农牧业发展规划，并在一定区域内实施。鼓励龙头企业加强原料基地建设，提升区域化布局、专业化生产、规模化经营水平。新增设施农业 2.2万亩，提高利用率和收益率。更加注重科学布局、技术支撑和市场营销，在临河、五原、杭后、磴口、前旗启动建设10个万亩以上集中连片设施农业园区， “十三五”时期达到35万亩。实施肉羊产业“三五”工程，新建规模化养殖场123个、种羊场55个。推进肉羊产业转型升级，加强饲养精细化管理，大力发展精深加工。继续支持河套酒业、二狼山白山羊绒等产业发展。加大“三品一标”认证支持力度，集中打造“河套”、“巴盟”、“乌拉特”等地域品牌。办好全国肉羊产业大会、绿色农畜产品进出口博览会、农牧业科技成果博览会。推进农牧业标准化和信息化，健全农畜产品质量安全监管体系、科技创新推广体系、社会化服务体系、动植物疫病防控体系和农畜产品流通营销体系。提高农牧业机械化水平。市本级安排1000万元专项资金，支持发展现代种业。加快发展水产养殖。依法有序推进土地流转。大力培养新型职业农牧民，引导大中专毕业生、返乡人员等领办合作社和家庭农牧场，发展多种形式适度规模经营。推动一二三产业融合发展，促进农牧业与电商、旅游、新能源、健康养老等良性互动，拓展延伸产业链和价值链。

(四)在稳工业抓项目促转型上下功夫。坚持提升传统产业与培育新兴产业并重，进一步做优存量、做大增量，提高工业经济质量效益。加强运行调度，做好为企业服务工作，“一企一策”帮助解决困难，促进企业扭亏减亏、恢复生产。协调金融机构推广“助保贷”业务，缓解企业流动资金紧张问题。全面落实支持企业发展的政策措施，提高企业投资信心，改善企业市场预期。开展质量品牌提升行动、“两化融合”贯标行动，引导企业提高产品技术、工艺装备和信息化水平。全年工业完成投资370亿元，实施千万元以上项目132项，其中亿元以上81项。加快推进海钰硫化工、东立多晶硅等项目建设，开工建设恒嘉200吨蓝宝石、铸合 800万吨银矿采选、太平矿业600万吨技改等项目，推动鑫型日处理100万立方米液化天然气、振森24万立方米纤维板等项目建成投产。加快乌中旗百万千瓦风电基地建设，科学合理配置风光资源，推动风电、光电向全产业链发展。发展农机制造、新材料、电子信息等产业，培育新的经济增长点。配套完善污水处理、铁路专用线等基础设施，增强园区承载能力。持续扩大对外开放，积极承接京津冀、环渤海等区域产业转移，主动融入呼包银榆经济区和沿黄沿线经济带，全方位开展招商引资。实施矿能产品专用通道等项目，推动跨境铁路、中蒙经济合作区建设工作。

(五)在培育壮大服务业上下功夫。实施“旅游+”行动，按照全域旅游理念加快开发精品旅游线路。推进黄河河套文化旅游区5A级景区创建工作，实施河套文化养生度假区、黄河古渡、酒庄老镇项目，建设跨包兰铁路通湿地公园人行桥、旅游公路等基础设施。推进纳林湖、多蓝湖、匈奴城、乌拉特部落等景区提档升级，实施太阳庙沙地、口岸国门和界碑旅游通道等项目。充分利用湖泊湿地众多的有利条件，大力发展冬季冰雪旅游。通过多种媒体，加大宣传营销力度，提高乡村旅游影响力和知名度，努力在全国占有一席之地。全年接待游客400万人次以上，旅游总收入突破40亿元。加快发展金融业，引进国泰君安证券、内蒙古银行等金融机构，新增20家企业进入资本市场融资。大力发展普惠金融，为小微企业、农村牧区提供方便快捷的金融服务。完善陆港基础设施，推动保税园区审批和临河铁路物流中心建设。健全城乡农贸市场和物流配送体系，支持企业在重点城市建立巴彦淖尔特色产品展销中心。扶持和推动电子商务健康发展，提升肉羊、煤炭、葵花籽等电子交易平台建设运营水平，探索建立河套农畜产品电子交易中心，加快推动电商进农村牧区。激发消费潜力，培育养老、家政、信息、文体等消费，引导消费朝着智能、绿色、健康、安全方向转变。加强外贸转型升级示范基地建设，巩固扩大番茄、籽仁、脱水菜等农畜产品出口市场。

(六)在完成“十个全覆盖”工程上下功夫。按照“不漏一村、不漏一户、不漏一人”的要求，紧盯薄弱环节，加大攻坚力度，同步推进精准扶贫、产业发展、乡风文明和基层党组织建设，年内规划保留的村庄基本实现全覆盖。因地制宜实施建设改造，保持乡村风貌和田园风光，体现水、绿、文化特色，把工程做精、做细、做美。加大财政投入力度，通过项目整合、金融贷款等形式，多渠道筹措建设资金。加强项目资金和工程质量监管，严格履行招投标程序，规范财务管理，坚决杜绝腐败现象发生。积极争取政策支持，稳步实施“土改砖”试点工作。配套建设公厕、污水、垃圾处理等设施，抓好“五堆”清理。完成1490个村庄绿化，积极申办全国村庄绿化现场会。建立以旗县区为单位的城乡环境卫生一体化管理机制，切实维护工程建设成果。组织干部下乡驻村，发动群众广泛参与。加强指挥调度，采取督查指导、巡回检查等措施，精准有效解决问题。严格考核验收，按照自治区党委的要求对7个旗县区进行排名，排名最后的问责追责。

(七)在深入推进新型城镇化上下功夫。城市工作在全局工作中具有举足轻重的地位。要始终坚持以人民为中心的发展思想，自觉尊重城市发展规律，全面落实 “五个统筹”的要求。立足市情，尊重自然、顺应自然、保护自然，改善城市生态环境，着力提升城市发展的持续性、宜居性。科学搞好城市规划，突出地域和文化特色，贯彻适用、经济、绿色、美观的建筑方针，把好山好水好风光融入城镇。编制完成中心城区近期建设和地下空间开发利用规划。全面加强城镇建设，坚持地上、地下设施一起抓，新区建设、旧城改造一起抓，不断完善“五城同创”硬件设施，提高城市的综合承载能力，促进中心城区、县城、集镇联动发展，让广大群众在家门口分享城镇化成果。实施绿化提档升级工程，新增园林绿化1700亩。新改建道路32公里，完成背街小巷治理30万平方米，建设各类管网158公里，新增集中供热面积200万平方米、天然气入户7800户，开展城市生活垃圾分类和餐厨废弃物资源化利用。推进双河新区建设，完成先锋新桥、敕勒大街项目，启动先锋南路、云中大街和美术学院新校区建设。稳步实施平房区基础设施配套改造，完成棚户区改造9954套。抓好23个重点集镇建设。切实加强城市管理和服务，以老百姓满意不满意、生活方便不方便为标准，努力提供精细化管理和良好的公共服务，下大力解决公共交通不便、物业服务水平不高等突出问题，推进城市管理向网格化、智能化、人性化方向发展，让城市的天更蓝、水更清、空气更清新，让群众生活得更舒心、更美好、更安全。

(八)在加强生态环境保护和基础建设上下功夫。坚持绿色发展、绿色惠民，倡导绿色生产生活方式，加快建设美丽巴彦淖尔。按照“三线”、“四区”布局，严格国土空间用途管制，做好生态红线划定工作，严禁在保护区探矿采矿。落实好新一轮草原生态补奖政策，持续实施禁牧休牧轮牧和草畜平衡。推进乌兰布和沙漠治理与保护，合理有效开发沙漠资源，新增沙产业5万亩。争取乌梁素海列入国际湿地名录，实施水循环、底泥无害化处置等项目，持续改善湖区水质。推进湿地保护与恢复工程，建立湿地资源管理档案。依托京津风沙源治理等项目，开展大规模国土绿化行动，完成林业生态建设60万亩。抓好机场绿化，提升绿化档次和水平，加快建设森林机场。加强林业病虫害防治和林木保护。大力发展循环经济，完成淘汰落后产能任务。深入开展大气、水、土壤污染防治，加强环境监管能力建设，严格环保执法。启动城镇污水处理厂提标、扩容和中水回用工程，实现稳定运行、达标排放。强化农村面源污染治理，推动秸秆、农膜等农牧业废弃物资源化利用。围绕补短板、强基础，提高投资有效性，加大基础设施投资力度。完成临哈高速主体工程，加快建设甘其毛都至临河一级公路，启动省道215、311改建工程，实施通村公路和街巷硬化2800公里。完成黑柳子、巴音杭盖等22项输变电项目。全年完成水利投资20亿元，实施黄河二期堤防、灌区续建配套与节水改造、农村牧区安全饮水提质增效等工程。

(九)在办好惠民实事上下功夫。市和旗县区财政安排民生专项支出26.9亿元，增加2.8亿元。打好脱贫攻坚战，通过发展生产、易地搬迁、生态补偿、发展教育、社会保障兜底“五个一批”措施，年内实现1.6万以上贫困人口稳定脱贫。市本级安排扶贫专项资金3000万元。以山旱区、乌兰布和沙区、乌梁素海周边地区为主战场，依托“三到村三到户”、金融扶贫富民等扶贫工程，实施精准扶贫、精准脱贫，因村因户因人施策，定点定人定责帮扶，支持贫困群众发展庭院经济，提高扶贫质量。坚持应保尽保、公平公正，完善最低生活保障制度。更加注重对特定人群、特殊困难的精准帮扶，加强农村牧区五保户、孤残人员救助，做好“救急难”试点工作，保障困难群众基本生活。推广“周末妈妈”等关爱活动，完善农村牧区留守儿童和妇女、老人关爱服务体系。建设保障性住房1.4万套，推进公租房社会房源收储配租试点。新建农村牧区互助幸福院3所，启动市失能失智老人呵护中心、民政精神病院扩建项目，加快市强制隔离戒毒所建设。全面落实困难、重度残疾人专项福利补贴政策。实施全民参保登记计划和城乡居民大病保险制度，简化新农合报销程序，加快社保卡、健康卡推广应用，改革医保支付方式。健全城镇职工参加养老保险持续缴费机制，提高生育保险医疗待遇。做好“三牧”工作，拓宽牧民就业增收渠道，大力实施兴边富民行动，落实边民补贴机制，改善牧民生产生活条件。

(十)在统筹推进社会建设上下功夫。新建改建幼儿园19所，建成义务教育标准化学校26所，推动乌中旗、杭锦后旗通过国家义务教育均衡发展验收。完成市直民族教育园区二期工程。建成河套学院新校区，部分院系实现搬迁。启用新区市医院，建设老年病康复医院。加强基层医务人员培养，扶持蒙中医药事业发展。全面落实一对夫妇可生育两个孩子政策，预防人口出生缺陷，提高出生人口质量。开展应对人口老龄化行动，加快慈善事业发展，维护妇女儿童、职工和残疾人的合法权益。大力推进以创建全国文明城市为引领的“五城同创”活动，推动创建工作常态化健康发展，集中力量解决一批事关民生和发展的重大问题。学习传统文化，弘扬中华传统美德，践行社会主义核心价值观，引导群众讲文明、知感恩、懂礼仪、重学习。办好沿黄河公路自行车赛、河套湿地龙舟赛、镜湖马拉松赛、“绿原仙境”摄影展等文体活动，开拍电视剧《鸿雁》，创作一批反映地方文化特色的歌舞剧、文学、艺术作品。加强对蒙俄及港澳台地区的文化交流，推动阴山岩刻申遗取得突破。倡导全民阅读，建设学习型社会。促进传统媒体与新兴媒体融合发展，提升巴彦淖尔新闻网、“掌上巴彦淖尔”、“河套微传媒”等新媒体影响力，加强对内对外宣传，做好舆论引导工作。推进足球改革发展，建成一批自治区和市级足球训练基地，打造12支高水平校园足球队。加强城乡社区建设，在农村牧区新建 14个社区。积极创新社会治理，建设平安巴彦淖尔，确保“五个不发生”。完善以信息化为支撑的社会治安立体防控体系，加强应急反恐处突力量和基础建设。全面推行“三化四全五确保”社会治理工作法，实施“三联六调、司法确认”社会矛盾纠纷排查预警和调处化解模式，加强和改进信访工作。严格安全生产和食品药品产品质量监管，坚决遏制重特大事故发生。全面贯彻党的民族政策，依法管理宗教事务，推动军民融合发展，加强爱民固边和军警民稳边固边建设，巩固民族团结、边疆安宁的局面。

各位代表，站在新起点，谋划新发展，落实新任务，必须加强政府自身建设，努力打造法治政府、务实政府、廉洁政府。坚持依法行政，认真落实《法治政府建设实施纲要》，把政府活动全面纳入法治轨道。自觉接受人大依法监督和政协民主监督，积极配合人大开展科学立法，认真组织实施政府协商计划。提高人大代表建议、政协委员提案及社情民意办理质量。市政府常务会议涉及民生工作、社会关注度高的议题，邀请人大代表和政协委员列席。启动新型智库建设，提高决策科学化水平。支持和保障法院、检察院依法独立公正行使审判权和检察权，广泛听取民主党派、工商联、无党派人士和人民团体的意见建议，注重发挥工会、共青团、妇联等群团组织作用，充分调动各方面积极性。深入落实中央八项规定和国务院“约法三章”，严控一般性支出和“三公”经费，6月底前基本完成公务用车制度改革。加强行政监察和审计监督，健全督查激励问责机制，坚决整治不作为、乱作为。严守党的政治纪律和政治规矩，严格执行廉洁自律准则和纪律处分条例，严肃查处违纪违法案件，不断加强廉政建设。政府公职人员要深入践行“三严三实”要求，增强新常态下履职尽责的能力，以优异的成绩赢得全市各族人民的信任。

各位代表，梦想承载希望，奋斗开创未来。让我们更加紧密地团结在以习近平同志为总书记的党中央周围，在自治区党委、政府和市委的坚强领导下，大力弘扬总干精神，胸怀中国梦，集聚正能量，提振精气神，真抓实干，激情创业，为夺取巴彦淖尔全面建成小康社会决胜阶段的新胜利而努力奋斗!
